# Adolescent Vision Health During the Outbreak of COVID-19: Association Between Digital Screen Use and Myopia Progression

**DOI:** 10.3389/fped.2021.662984

**Published:** 2021-05-25

**Authors:** Ji Liu, Baihuiyu Li, Yan Sun, Qiaoyi Chen, Jingxia Dang

**Affiliations:** ^1^Faculty of Education, Shaanxi Normal University, Xian, China; ^2^School of Basic Medical Sciences, Xian Jiaotong University, Xian, China; ^3^The First Affiliated Hospital, Xian Jiaotong University, Xian, China

**Keywords:** COVID-19, myopia–epidemiology, screen use, adolescents, children health risk

## Abstract

The coronavirus (COVID-19) pandemic has impacted education systems globally, making digital devices common arrangements for adolescent learning. However, vision consequences of such behavioral changes are not well-understood. This study investigates the association between duration of daily digital screen engagement and myopic progression among 3,831 Chinese adolescents during the COVID-19 pandemic. Study subjects report an average of 2.70 (*SD* = 1.77), 3.88 (*SD* = 2.23), 3.58 (*SD* = 2.30), and 3.42 (*SD* = 2.49) hours of television, computer, and smartphone for digital learning use at home, respectively. Researchers analyzed the association between digital screen use and myopic symptoms using statistical tools, and find that every 1 h increase in daily digital screen use is associated with 1.26 OR [Odds Ratio] (95% CI [Confidence Interval: 1.21–1.31, *p* < 0.001]) higher risks of myopic progression. Using computers (OR = 1.813, 95% CI = 1.05–3.12, *p* = 0.032) and using smartphones (OR = 2.02, 95% CI = 1.19–3.43, *p* = 0.009) are shown to be associated with higher risks of myopic progression than television use. Results from additional sensitivity tests that included inverse probability weights which accounted for heterogeneous user profile across different device type categories confirm that these findings are robust. In conclusion, this study finds that daily digital screen use is positively associated with prevalence of myopic progression and holds serious vision health implications for adolescents.

## Introduction

The global outbreak and spread of the coronavirus disease 2019 (COVID-19) has had a tremendous impact on adolescents around the world. During the peak months of the pandemic, 192 countries/territories elected to close schools, affecting nearly 1.5 billion children and young people (1). In consideration of youth safety, health, and well-being, a clear policy imperative has been centered around leveraging digital learning as a measure of remediation to reduce adverse impacts of school interruption. While a combination of remote learning and digital technology presents a timely solution to mitigate school closures, emerging research suggests that extended exposure to electronic devices and digital screens during the COVID-19 pandemic can have consequential impact on vision development for adolescents (2). Most strikingly, adolescence is a critical stage for sensory function development and a period characterized by intensive eye use among school-age children (3). In fact, prior to the COVID-19 outbreak, there was already widespread concern regarding a looming global youth vision crisis. According to the World Health Organization, at least 2.6 billion people worldwide suffer from impaired vision, among which a significant proportion are those under 18 years old (4). It is estimated that by 2050, 5.7 billion people or 59.6% of the world's population will become myopic (5), affecting close to one-half of the world's total population (6).

While myopia (near-sightedness) is often categorized as a benign disorder, its widespread prevalence among youths has been growing at an alarming rate (7), and the associated risks of severe vision impairment pathologies, including macular degeneration, posterior staphyloma, retinal detachment, cataract, and glaucoma, raise serious global public health concerns (8). On the one hand, studies have shown that myopia is closely related to individual traits, such that it is significantly more prevalent among girls (9), older children (10), and those from urban households (11). On the other hand, research has shown that myopic progression is influenced by environmental and lifestyle factors, particularly highlighting the critical role of prolonged proximate digital screen use (12). Notably, East Asia has been particularly affected by increased prevalence of myopia and early age of onset, due to rising school pressure, digitization of instruction, and consequent lifestyle changes (13). The prevalence of adolescent myopia in regional cities such as Hong Kong SAR, Singapore, and Tokyo has increased rapidly over the past 50–60 years (14), and in some cases with 80–90% of high school students being myopic and 10–20% highly myopic (15). In stark comparison, only 10–20% of the Chinese population was nearsighted 60 years ago, but myopia has been found to affect nearly 90% of Chinese teenagers in 2015 (16).

The introduction of virtual learning tools during the COVID-19 pandemic and the consequent rapid rise in screen time use may serve as an influential exogeneous shock that could propel higher incidences of myopia by re-shaping instructional and behavioral changes conducive to the onset and progression of myopic vision disorders. At the physiological level, near-vision eye use has been found to cause ciliary muscles to thicken (17) and is associated with increased refractive power of the retina, elongated eye axis, and myopic vision disorder (18). To this end, the adolescent eye is not yet fully developed and near-vision electronic display stimulation can result in extended exposure to hyperopic defocus. Studies have suggested chronic peripheral hyperopic defocus triggers compensating axial myopic eye growth, which alters refractive vision development prematurely (19).

At the population level, digital screen use has been identified as a leading risk factor causing vision disorder among children (20). Importantly, eye strain and fatigue symptoms occur with as little as 60 min of smartphone use (21), and duration of computer use among children is positively associated with vision disorder progression (22). In a study of Danish youths, it was found that digital screen use explains about 25% of the observed prevalence of myopia, with heightened myopic risks if digital screen use was more than 6 h per day (23). In another study in India, researchers concluded that screen time >2 h per day was positively associated with rates of myopic progression, and the effect of screen time was more salient in areas with lower prevalence of myopia (24). More specifically in China, 60 min of computer use per day was found to be significantly associated with increased myopia incidence among school-age children (25).

However, some researchers also point out that conclusive evidence on the link between digital screen use and myopic vision disorder among adolescents is still lacking in the literature (26). For one, there is only a handful of studies investigating this topic among recent age cohorts among youths who are exposed to significantly more digital screen use compared with previous age cohorts (27). For another, there might not have been enough contrast in terms of screen time exposure for the same individual, since adoption of digital devices among children commonly follow a stable and gradual trajectory (28). To add, children nowadays have access to a wide range of electronic devices which have varying screen sizes and are viewed at different distances; yet to the best of our knowledge, there exists no study at present that focuses on how device type influences vision impairment among young people.

In broad strokes, this study aims to fill this void in existing research by leveraging a nationwide experiment in China to implement remote learning and instruction for all school-age children, which expectedly result in substantial increase in digital screen use during the recent COVID-19 outbreak. More concretely, this study seeks to answer three interrelated research questions. First, how does digital screen time and myopic progression differ by individual traits? Second, what is the association between digital screen time and myopic progression? Third, how does digital device type influence myopic progression?

## Methods

### Subjects and Sampling

We collaborate with a nationally-known education-focused newspaper *Teachers Daily* to conduct an online survey to assess youth vision problems. The questionnaire was distributed nationally from May 12 to May 18, 2020 via *Teachers Daily*'s school networks which solicited student responses from 29 provinces and autonomous regions through the *Wenjuanxing* platform https://www.wjx.cn. Completing the standardized questionnaire takes about 10–15 min online. The inclusion criteria for participants are as follows: (1) can read and understand the Chinese questionnaire; (2) currently enrolled in pre-primary, primary, lower-secondary, or upper-secondary schools; (3) volunteered to participate in the survey; (4) submitted only one response using the same IP address; (5) whose guardians have completed the informed consent form. A total of 3,831 respondents from kindergarten to 12th grade satisfied the inclusion criteria. This study was approved by the Institutional Review Board of Shaanxi Normal University, and abided by ethics code of the World Medical Association Declaration of Helsinki.

### Measures and Variables

The standardized questionnaire contained detailed individual background information such as sex, grade-level, urban-rural status, as well as responses on prior and current vision condition, length of digital screen use per day since COVID-19 outbreak, and type of digital device used. Importantly, the questionnaire prompted respondents to evaluate the present state of their eyesight. Respondents were asked to self-evaluate symptomatic changes in their near-vision condition using the Lay Terms Approach, which prompts adoption of terminologies that subjects are familiar with (29), such as whether they experience blurry vision when viewing distant objects since beginning remote learning and instruction. Finally, for classification of digital device type, we collect respondents' digital screen usage information according to different size categories: large (television), moderate (personal computer), small (smartphone), as well as if respondents use a combination of multiple digital devices.

### Statistical Analysis

The dataset was prepared and analyzed using STATA version 15.0 (Stata, StataCorp LLC, College Station, TX). First, we conduct descriptive statistical analysis, and report paired sample *t*-test, *f*-test, and chi-square test results assessing to what extent digital screen time and self-reported incidence of myopic symptoms differ by individual traits. Second, we fit a binary multivariate logistic regression model to examine the association between digital screen time and self-reported myopia progression, after controlling for individual traits and pre-pandemic vision condition. The dependent variable is self-reported “Myopic Symptoms” (No = 0, Yes = 1) and the key explanatory variable “Daily Digital Screen Time” is reported in hours. Individual-level demographic control variables include sex (male = 0, female = 1), pre-primary (otherwise = 0, preprimary = 1), primary (otherwise = 0, primary = 1), lower-secondary (otherwise = 0, lower-secondary = 1), with upper-secondary being the omitted reference grade-level, and the variable urban-rural transitional (otherwise = 0, urban-rural transitional = 1), rural (otherwise = 0, rural = 1), with urban as the omitted reference category. Pre-pandemic vision condition (otherwise = 0, myopic = 1) is also recorded. Third, we include digital screen type variables, which are reported as computer (otherwise = 0, computer = 1), smartphone (otherwise = 0, smartphone = 1), multiple devices (otherwise = 0, more than 1-device = 1), with television as the omitted reference category. As sensitivity test, we also calculate inverse probability weights by device type, since user profile across device type categories is expected to be non-randomly heterogeneous. Inverse probability weighting, which is a common statistical technique adopted to adjust for non-random selection in observational studies (30), is estimated from a binary logistic regression model predicting subject's device type of choice for digital learning engagement, and includes all individual-level variables previously stated. Critical-α level of significance is set at *p* < 0.05 (two-sided) for all statistical analyses.

## Results

As shown in Table 1, a total of 3,831 respondents satisfied the inclusion criteria and form the analytical sample. Of the included respondents, 1,973 (51.5%) are male, 1,858 (48.5%) are female, with 426 (11.1%) attending pre-primary education, 2,234 (58.3%) in primary, 269 (7.1%) in lower-secondary, and 902 (23.5%) in upper-secondary schools. Among the respondents, 2,925 (76.4%) are located in urban areas, 271 (7%) reside in urban-rural transitional areas, and 635 (16.6%) live in rural areas. As for prevalence of myopic condition prior to the COVID-19 pandemic, 2,440 (63.7%) respondents report no issues, whereas 1,291 (36.3%) report existing myopic condition. In terms of device type used for remote learning during the COVID-19 pandemic, 93 (2.4%) respondents use television, 735 (19.2%) use computers, 2,193 (57.2%) use smartphones, and 810 (21.2%) report using multiple devices.

**Table 1:** Prevalence of myopic symptoms and duration of daily digital screen engagement by individual traits (*N* = 3,831).

**Variables**	***n***	**%**	**Daily digital screen time (hours)**			**Myopic symptoms (No** **=** **0, Yes** **=** **1)**		
			**Mean**	**SD**	***p*-value**	***n***	**%**	***p*-value**
**Sex**								
Male	1973	51.5	3.51	2.36	0.041^a^	727	36.85	0.272^c^
Female	1858	48.5	3.66	2.29		717	38.59	
**Grade**								
Pre-Primary	426	11.1	1.30	0.86	<0.001^b^	86	20.19	<0.001^c^
Primary	2234	58.3	2.90	1.87		711	31.83	
Lower-Secondary	269	7.1	4.89	2.03		135	50.19	
Upper-Secondary	902	23.5	5.96	1.72		512	56.76	
**Location**								
Urban	2925	76.4	3.64	2.36	<0.001^b^	1131	38.67	0.060^c^
Urban-Rural transitional	271	7.0	3.69	2.10		99	36.53	
Rural	635	16.6	3.28	2.36		214	33.70	
**Pre-pandemic myopia**								
No	2440	63.7	2.82	2.02	<0.001^a^	636	26.07	<0.001^c^
Yes	1391	36.3	4.93	2.21		808	58.09	
**Device type used**								
Television	93	2.4	2.70	1.77	<0.001^b^	20	21.51	<0.001^c^
Computer	735	19.2	3.88	2.23		289	39.32	
Smartphone	2193	57.2	3.58	2.30		864	39.40	
Multiple devices	810	21.2	3.42	2.49		271	33.46	

a *p-value based on T-test*,

b *p-value based on F-test*,

c *p-value based on Chi-square test*.

The associational relationship with duration of digital screen engagement during the COVID-19 pandemic is statistically significant (*p* < 0.001) across all individual trait measures. First, duration of digital screen engagement differs marginally (*p* = 0.041), with females reporting slightly more hours of daily digital screen use. Second, digital screen time is shown to be statistically different (*p* < 0.001) among grade-levels, with higher grade-levels reporting substantially more hours of additional digital screen time. Third, respondents from both urban and urban-rural transitional areas report significantly (*p* < 0.001) more hours of digital screen engagement than respondents in rural areas. Fourth, those respondents who self-identify as myopic in pre-pandemic times, report larger (*p* < 0.001) increase in digital screen use duration than those who are not. Fifth, additional digital screen time during the COVID-19 pandemic differs significantly (*p* < 0.001) by device type, with those who use television reporting the least number of hours.

As shown in the final three columns of Table 1, differences in prevalence of myopic symptoms during the COVID-19 pandemic are statistically significant (*p* < 0.001) for some variables but not others. On the one hand, myopic progression during the COVID-19 pandemic does not differ meaningfully between males and females (*p* = 0.272), or among urban, urban-rural transitional, and rural locations (*p* = 0.060). On the other hand, there are significantly more (*p* < 0.001) individuals reporting myopic symptoms from pre-primary, lower-secondary, and upper-secondary grades, while respondents enrolled in primary grades report significantly less so. In addition, respondents who are already myopic prior to the COVID-19 pandemic tend to report myopic progression at higher rates (*p* < 0.001) than those who did not self-identify as suffering from myopia in pre-pandemic times. Importantly, respondents who report using television for remote learning indicate significantly less (*p* < 0.001) degree of myopic progression, as compared to other device types.

Table 2 presents results from binary multivariate logistic regression which analyzed the degree to which various influencing factors affect myopic progression. Most strikingly in the multivariate logistic regression, every additional hour in digital screen time is associated with 1.26 OR [Odds Ratio] (95% CI [Confidence Interval: 1.21–1.31]) higher risks of myopic progression. Results also indicate that pre-COVID myopia condition (OR = 2.74, 95% CI: 2.32–3.23) is associated with higher risks of myopic progression, while using computers (OR = 1.813, 95% CI = 1.05–3.12) or using smartphones (OR = 2.02, 95% CI = 1.19–3.43) both exhibit higher risks of myopic progression, as compared to using television as remote learning medium. The final three columns in Table 2 also include results from the sensitivity analysis by performing inverse probability weighting by device type. Inverse probability weights are estimated from a binary logistic regression model predicting subject's device type of choice, and includes all variables in the first column of Table 2. Once inverse probability weights are included, the results, as shown in the final three columns of Table 2, are qualitatively similar to that in the unweighted regression. Altogether, sensitivity test results indicate that user profile heterogeneity which vary by device type does not significantly sway key findings, and support the conclusion that duration of digital screen engagement, smartphone use, and computer use are positively associated with myopia progression, respectively.

**Table 2:** Binary multivariate logistic regression analysis on influencing factors of myopic progression (*N* = 3,831).

**Variables**	**Multivariate logistic regression**			**Multivariate logistic regression, inverse probability weighted**		
	**OR**	**95% CI**	***p*-value**	**OR**	**95% CI**	***p*-value**
Daily digital screen Time (h)	1.26	1.21–1.31	<0.001	1.30	1.22–1.38	<0.001
**Sex**						
Male	1			1
Female	0.99	0.86–1.14	0.993	1.16	0.89–1.52	0.276
**Grade**						
Pre-primary	1.14	0.80–1.62	0.476	1.25	0.61–2.60	0.547
Primary	1.14	0.91–1.42	0.247	1.22	0.84–1.78	0.289
Lower-Secondary	1.06	0.79–1.42	0.681	1.58	0.79–3.15	0.197
Upper-Secondary	1			1
**Location**						
Urban	1			1
Urban-Rural transitional	0.91	0.69–1.20	0.488	0.79	0.54–1.15	0.217
Rural	0.97	0.79–1.17	0.713	1.25	0.80–1.94	0.327
**Pre-pandemic myopia**						
No	1			1
Yes	2.74	2.32–3.23	<0.001	2.33	1.72–3.16	<0.001
**Device type**						
Television	1	
Computer	1.81	1.05–3.12	0.032	1.57	1.10–1.98	0.010
Smartphone	2.02	1.19–3.43	0.009	1.74	1.27–1.89	0.005
Multiple devices	1.56	0.90–2.68	0.111	1.37	0.77–2.42	0.274

*OR, odds ratio; CI, confidence interval*.

## Discussion and Conclusions

To the best of our knowledge, this study is among the first to examine the association between digital screen time and myopic progression among a full age-spectrum of adolescents, from pre-primary to upper-secondary, in the context of a nationwide remote learning experiment during the COVID-19 outbreak in China. Using a large-scale national survey, we present four main findings. First, we find that digital screen use duration has likely sharply increased during the COVID-19 pandemic as result of widespread school closures, and the duration of digital screen engagement is heterogeneous by sex, grade, location, pre-pandemic vision condition, and device-type used. Second, myopic symptom is more prevalent among respondents from upper-secondary and lower-secondary grades, and among those who report being myopic prior to the COVID-19 pandemic. Third, and most importantly, duration of digital screen engagement is positively associated with higher risks of symptomatic myopic progression. Fourth, results show that respondents who use computers and smartphones, as opposed to using television as mode of remote distance learning, show markedly higher likelihood of myopic progression.

We build on prior studies that examine the association between digital screen time and myopic vision progression among adolescents (17–25), by leveraging an arguably exogeneous COVID-19 pandemic-induced remote digital learning experiment. On the one hand, our results show that duration of digital screen engagement among Chinese adolescents has been substantial as result of COVID-19 pandemic-related school closures. Recent studies have echoed our findings and called on both policy and clinical attention to the lesser visible yet influential consequences of the pandemic, especially on adolescent health (2, 12, 31, 32). Furthermore, our results contribute to emerging evidence on the link between digital screen use and myopic vision impairment (33–35). Consistent with a recent longitudinal study on Chinese youths (36), our results show that increased digital screen exposure is positively associated with higher risks of myopia. More strikingly, our study is contextually situated during a critical period of time characterized by widespread school closures at the height of the COVID-19 pandemic, and is among the first to identify heterogeneous effects that exist among different digital device types. Prior studies have not independently assessed how variation in device type can affect myopic progression among adolescents (27).

Based on our findings, we speculate that digital devices with larger screens allow users to view and access digital contents at a reasonable distance, avoiding intensive near-vision eye use and reducing subsequent hyperopic defocus stimulation, while digital devices equipped with smaller screens require viewing at arms-length or less, which may increase the likelihood of myopic symptom onset and progression (21). In this regard, a potentially useful clinical implication is to consider providing recommended digital screen viewing distance information and duration of device use reminder interventions to youths and their families, particularly during next phases of COVID-19 pandemic-related remote learning programs.

Notwithstanding, a limitation in this study worth mentioning is the use of self-reported vision condition, instead of relying on specialist examinations. The decision to implement self-reported myopic measures are in two-folds. First, self-reported measures allow for rapid survey rollout and large-scale population coverage. This is especially important during the COVID-19 pandemic, as face-to-face specialist eye examinations are not viable in consideration of safe distancing measures and closure of optometry clinics. Second, prior research has indicated that detailed ophthalmic evaluation and self-reported refractive error responses do not differ systematically, and has recommended utilizing self-report as a reasonably accurate alternative approach to elicit refractive status in population-based questionnaires with large sample sizes (37). In addition, while we cannot safely rule out the confounding influence of reduction in outdoor activities on the association between digital screen use and myopic progression (38), this study's setting is unique because variation in outdoor activities is likely minimized during the COVID-19 outbreak in China due to extensive outdoor and public curfew measures (39).

In conclusion, our findings suggest that pandemic-induced school closures and subsequent remote learning arrangements have resulted in extended duration of daily digital screen engagement among Chinese adolescents, and that risks of myopic symptom onset and progression increase with every additional daily hour of digital screen engagement. Our study cautions against the adverse health consequences of widespread digital learning rollout during the COVID-19 pandemic, and highlight the importance of channeling learning opportunities through a variety of instructional media, upon considering the heterogeneous degrees of influence that different devices can have on vision health. Efforts should be made to encourage students to limit their digital screen engagement in order to avoid heightened risks of vision impairment, especially for youths who continue to be affected by the COVID-19 pandemic and those who must resort to remote learning arrangements.

## Data Availability Statement

Restrictions apply to the availability of data used, due to study subject privacy protection. Data was obtained from Teachers Daily, and is available upon request with the permission of Teachers Daily.

## Ethics Statement

The studies involving human participants were reviewed and approved by Shaanxi Normal University. Written informed consent to participate in this study was provided by the participants' legal guardian/next of kin.

## Author Contributions

JL, QC, and JD conceived, conceptualized, and designed the study. JL and QC contributed to data collection and conducted the statistical analysis. JL, BL, YS, and QC drafted the article and contributed to interpretation of results. All authors have revised the manuscript for important intellectual content and have read and approved the final manuscript.

## Conflict of Interest

The authors declare that the research was conducted in the absence of any commercial or financial relationships that could be construed as a potential conflict of interest.
